# From ‘My world fell apart’ to ‘The best thing that happened in my life’: elite athletes’ longitudinal journeys after an injury

**DOI:** 10.1136/bmjsem-2026-003320

**Published:** 2026-09-25

**Authors:** Caroline Bolling, Richard Weiler, Jonathan T Finnoff, Gino MMJ Kerkhoffs, Jonahnnes L Tol, Kay M Crossley, Francesco Della Villa, Vincent Gouttebarge, Evert Verhagen

**Affiliations:** 1 Amsterdam UMC location University of Amsterdam, Department of Orthopedic Surgery and Sports Medicine, Academic Center for Evidence Based Medicine, Amsterdam IOC Center ACHSS, Amsterdam, The Netherlands; 2 Amsterdam Collaboration on Health & Safety in Sports, Department of Occupational Health, Amsterdam Movement Sciences, Amsterdam UMC, The Vrije Universiteit Amsterdam, Amsterdam, Netherlands; 3 Institute Sport, Exercise and Health, Division of Surgery and Interventional Science, UCL, London, UK; 4 Fortius Clinic, London, UK; 5 US Olympic & Paralympic Committee, Colorado Springs, Colorado, USA; 6 Department of Physical Medicine and Rehabilitation, University of Colorado School of Medicine, Aurora, Colorado, USA; 7 US Coalition for the Prevention of Illness and Injury in Sport, Colorado Springs, CO, USA; 8 University of Utah Department of Physical Medicine and Rehabilitation, Salt Lake City, UT, USA; 9 Amsterdam Movement Sciences, Amsterdam, The Netherlands; 10 Aspetar, Orthopedic and Sports Medicine Hospital, Doha, Qatar; 11 La Trobe Sport and Exercise Medicine Research Centre, La Trobe University, Melbourne, Victoria, Australia; 12 Australian IOC Research Centre, La Trobe University, Melbourne, Victoria, Australia; 13 Education and Research Department, FIFA Medical Centre of Excellence, Isokinetic Medical Group, Bologna, Italy; 14 Section Sports Medicine, Faculty of Health Sciences, University of Pretoria, Pretoria, South Africa; 15 Medical & Anti-Doping, Football Division, Union of European Football Association, Nyon, Switzerland

**Keywords:** Qualitative Research, Sports rehabilitation programs, Sports medicine, Sports physiotherapy, Athlete

## Abstract

This study explored the perspectives of elite athletes diagnosed with an injury requiring a minimum of 4 months of rehabilitation based on the health professional’s prognosis. Through a longitudinal phenomenological design, we captured perceptions and experiences of elite athletes from different sports, countries and injury types following major injury. Athletes were interviewed at different stages of the return to sport process (from early rehabilitation to return to performance), resulting in a total of 31 interviews analysed using longitudinal interpretative phenomenological analysis. Three overarching journey phases were identified. (1) The Rough Start was characterised by uncertainty about the future, loss of athletic identity, emotional distress and feelings of isolation as athletes adjusted to injury. (2) The Roller coaster rehab reflected emotional fluctuations, small achievements and setbacks, the influence of previous injury experiences, the importance of support systems and challenges related to communication with health professionals. (3) The Journey’s End involved athletes feeling like athletes again, experiencing a sense of accomplishment and reflecting on personal growth. During this phase, some athletes reassessed priorities or made decisions about career transitions. The final steps towards return to performance were often shaped by factors beyond athletes’ control, including team dynamics, competition opportunities and organisational decisions. Despite shared themes, athletes’ narratives showed substantial variability. This study provides in-depth insight into how athletes’ journeys are non-linear and highly individual. It highlights the importance of flexible, athlete-centred care that values communication, individual responses to injury and the wider sporting and career context, rather than relying solely on standardised protocols or physical criteria.

**WHAT IS ALREADY KNOWN ON THIS TOPIC**

- Return to sport (RTS) is a comprehensive process aimed at safely reintegrating athletes into their sport after injury, considering physical and psychosocial aspects.
- The process is described as a continuum of different phases that focus on achieving clinical outcomes and functional improvement, based on objective criteria.
- It is an individualised process influenced by many contextual factors.

**WHAT THIS STUDY ADDS**

- Athletes’ perspectives evolve during the recovery journey phases, which influences their views, motivations, decisions and potentially clinical outcomes, emphasising the need for ongoing dialogue rather than one-off RTS decisions.
- Returning to performance is highly influenced by factors beyond the athlete’s and health professionals’ control, such as the timing of the season, contracts, competition calendars and team squad demands.
- The athlete’s recovery journey is influenced by the culture, structure, resources available to the sport and country, the injury history and prognosis and the social support, which changes their perception and coping strategies during the process.

**HOW THIS STUDY MIGHT AFFECT RESEARCH, PRACTICE AND POLICY**

- Health and performance professionals should adapt to the athlete’s changing and evolving needs for information, support, and guidance throughout the process.
- Clinicians should discuss with athletes the meaning of their objective criteria and expected of RTS, timing to as they can impact the athlete’s recovery journey and motivation.
- Communication flow needs to be coordinated to ensure aligned expectations and clear goal setting across all involved professionals.

## Introduction

 Return to sport (RTS) after injury is a core topic in the sports medicine literature.^1 2^ Returning safely and optimally to the previous level of performance is commonly considered the ultimate goal.^3^ The RTS process should be goal-oriented and criteria-based, with shared decision-making and acknowledgement of physical, psychological, social and contextual factors.^4–6^ The athletes should be empowered and engaged in the process.^7 8^ Objective criteria, such as functional tests, physical and psychological abilities measurements, are important to monitor progression and assess readiness to RTS.^9 10^ However, the RTS criteria are typically time-based, injury- or sport-specific and not sufficiently context-sensitive.^11^ RTS studies in elite sports are developed through a performance or health lens; however, longitudinal lived experiences are usually absent. In particular, there is a lack of longitudinal accounts that trace how elite athletes’ meanings, priorities and decisions shift across the RTS continuum.

We know that an injury occurs in an athlete, embedded within an individual’s biopsychosocial environment, participating in a sport shaped by a broader social structure in a specific place and time.^12^ Injuries have an expected healing trajectory and RTS timeline. However, athletes with similar injuries, playing levels and comparable care may experience vastly different rehabilitation journeys, even if they achieve the same outcome.^13 14^ When assessing athletes’ readiness to RTS, the same functional test results may be interpreted differently depending on factors such as the type of sport, level of play, the athlete’s experience, the availability of replacements or the timing within the competitive season.^2 15^ While clinical experience recognises the diversity of RTS experiences, responses and outcomes, our clinical guidelines and protocols are less flexible and rigidly prescriptive.

Beyond the physical healing process, psychological, social and contextual factors play a crucial role in shaping an athlete’s recovery journey.^6 16 17^ Psychosocial-related reasons are significantly more cited for failure to RTS than knee-related reasons.^18^ Athletes face emotional challenges such as fear of reinjury.^17 19 20^ At the same time, coping mechanisms, social support and the therapeutic alliance contribute to recovery success.^13 21^ Furthermore, broader contextual elements, such as sports culture, access to resources and environmental demands, can either facilitate or hinder progress.^22 23^ RTS process is complex and context-specific for each athlete; however, to better understand how to communicate and manage RTS, we must attempt to understand and listen to the athlete’s perspectives.^24 25^

While the literature on RTS phases and criteria is extensive, there is relatively little research on how the RTS process unfolds in real-life practice in elite sports. A few qualitative studies have retrospectively explored athletes’ experiences post-injury, providing insight into how athletes experienced the RTS process.^13^ However, various factors and circumstances evolve across recovery stages, and the perspective of athletes during the actual journey remains underrepresented. If RTS research principles need to be applied in real-life practice, then a deeper understanding of the athlete’s journey is needed. Therefore, we used serial qualitative interviews to explore elite athletes’ perspectives across the continuum from early rehabilitation to return to performance, capturing changes in perceptions over time during their RTS journey following a major injury.

## Methods

### Design

We followed a phenomenological approach, which focuses on understanding the essence of lived experience and analysing how individuals feel, sense, perceive and make sense of their world, making it well-suited to exploring evolving RTS journeys.^26 27^ This study was situated within an interpretivist paradigm and located within the broader phenomenological tradition. We understood RTS following long-term injury as a subjective and evolving experience situated within athletes’ personal, social and sporting contexts. Consistent with a hermeneutic epistemology, knowledge was considered co-constructed through participants’ accounts and the researchers’ interpretation of those accounts. We followed a Longitudinal Interpretative Phenomenological Analysis (LIPA) which does not rely on just one single school of phenomenology. Instead, LIPA aligns with standard IPA principles blending elements from multiple historical schools from Husserl and Heidegger and Gadamer.^28 29^ So, we drew on phenomenology as a broad methodological orientation to guide attention to participants’ subjective experiences and meaning-making processes.^30^ Given that RTS is a dynamic and evolving journey, we used serial interviews to capture changes over time and to develop a rich, contextualised account of athletes’ experiences.^28 31^

### Recruitment and participants

The participants were recruited through partners, including Olympic committees and health professionals. Potential participants had to be current athletes (actively competing at elite level at the time of injury), at least 18 years old, performing any Olympic sport and representing their country at the highest level in international competitions. They had to have been diagnosed with an injury requiring a minimum of 4 months of rehabilitation (based on the health professional’s recommendations). Participants had to be enrolled in the study within 1 month of the date of the injury or surgery. When a potential participant was identified by a healthcare partner, the research team was notified and provided with their contact information. The research team then contacted the potential participant, and after obtaining informed consent, enrolled them in the study and scheduled the first interview. This eligibility criteria made recruitment highly time-dependent and circumstance-dependent, so we relied on healthcare partners identifying eligible athletes at the right moment, which was inherently unpredictable. A total of nine athletes were invited. Two athletes agreed to participate when asked by a recruiting partner but did not respond to contact attempts by the researcher. Two athletes were found not to meet inclusion criteria after initial contact and were excluded: one athlete had been injured for 6 weeks and another was under the selected age. In such cases, the lead researcher communicated this sensitively and directly, explaining the reasons and providing an opportunity for questions. No further distress was observed in any case. A total of 5 athletes were included in the study. This small, information-rich sample enabled in-depth, longitudinal engagement with each athlete’s evolving journey. With five participants and 31 interviews, the richness and complexity of the individual stories confirmed that continuing to recruit additional cases would not meaningfully add to the conceptual depth already achieved. The average duration of interviews was 31 min and the recorded data totalled 16 hours and 8 min.

### Data collection

Serial interviews were conducted at different RTS stages. We aimed to interview the athletes at various time points, including after injury or surgery, during early-stage rehabilitation, mid-stage rehabilitation, on-field rehabilitation or sports-specific activities, return to training, return to competition (when the athlete went back to play) and return to performance (when the athlete went back playing their previous level of performance), based on the current literature.^3 32^ After the first interview, the subsequent interview was planned, considering the timing, goal-setting and upcoming planned activities described by the athlete. Before the interview, the researcher would contact the athlete and confirm if the timing was appropriate. The interviews explored how athletes perceive the process, their experiences during the journey, their emotional responses, perceptions of progress and the contextual factors that influence the process. The interview guide is included in online supplemental file 1). The interview guide served as a flexible framework. Subsequent interviews were individually tailored, incorporating elements from previous conversations with each athlete. Questions were adapted to the athlete’s current stage and circumstances, allowing the exploration of individual experiences to evolve over time. The lead researcher was actively and continuously engaged during data collection, revisiting notes and listening to previous interviews before each subsequent interview with the same athlete, which directly informed how those conversations developed and what threads were followed up. Online interviews facilitated the accessibility of participants and ensured the participation of an international sample. They took place on an online platform (Microsoft Teams or any other platform of the participant’s choice) and were audio-recorded.

### Data analysis

All interviews were transcribed verbatim using Descript (2025) and reviewed by the main researcher (CB). The analysis focused on identifying patterns while preserving the individuality of each athlete’s experience. We applied the recommended approach for conducting LIPA, paying attention to change over time within each case which is aligned with our aim to explore how athletes’ experiences, meanings and perceptions evolve across the RTS continuum. LIPA is designed for serial data, allowing the researcher to attend to change, development and continuity within individual cases over time, before engaging in cross-case analysis.^28^ The lead researcher constantly revisited the interviews during data collection and the systematic analysis started after all the interviews were completed. LIPA’s cross-case stage involves an interpretative synthesis of patterns of convergence and divergence in meaning across participants, while preserving sensitivity to the uniqueness of each trajectory. This process allows for the development of overarching concepts that capture shared meanings without losing the contextual specificity of individual cases.

MaxQDA (VERBI Software. MAXQDA 2022. Berlin: VERBI Software, 2021) was used to code and organise the data. The process began by treating each participant’s data as a unique case before engaging in cross-case analysis. Initially, the first author (CB) read and listened to the interviews in chronological order for each participant. Throughout this process, it was considered when in the RTS journey particular concepts occurred, strengthened or diminished to retain a longitudinal perspective. After reading and re-reading, CB created notes of observations and reflections, building a comprehensive picture of athletes’ stories. Subsequently, CB coded each interview, following the participants’ narrative. Each case was then analysed in detail to identify emergent patterns within the individual trajectory. Individual narrative summaries were developed based on similarities and noted recurring patterns on how meaning-making developed, shifted or persisted across time points developing concepts. These narratives and related concepts were then compared and supported by an interpretation following a cross-case analysis. Finally, an interpretative synthesis of patterns of convergence and divergence in meaning across participants identified overarching concepts that capture shared meanings without losing the contextual specificity of individual cases. The results structure was shared with authors EV and RW for feedback and subsequently circulated to the remaining authors. The collaborative interpretive discussions contributed to the rigour of the analysis by promoting reflexivity and transparency in analytic decision-making. Figure 1 provides an overview of the data analysis process.

**Figure 1:**
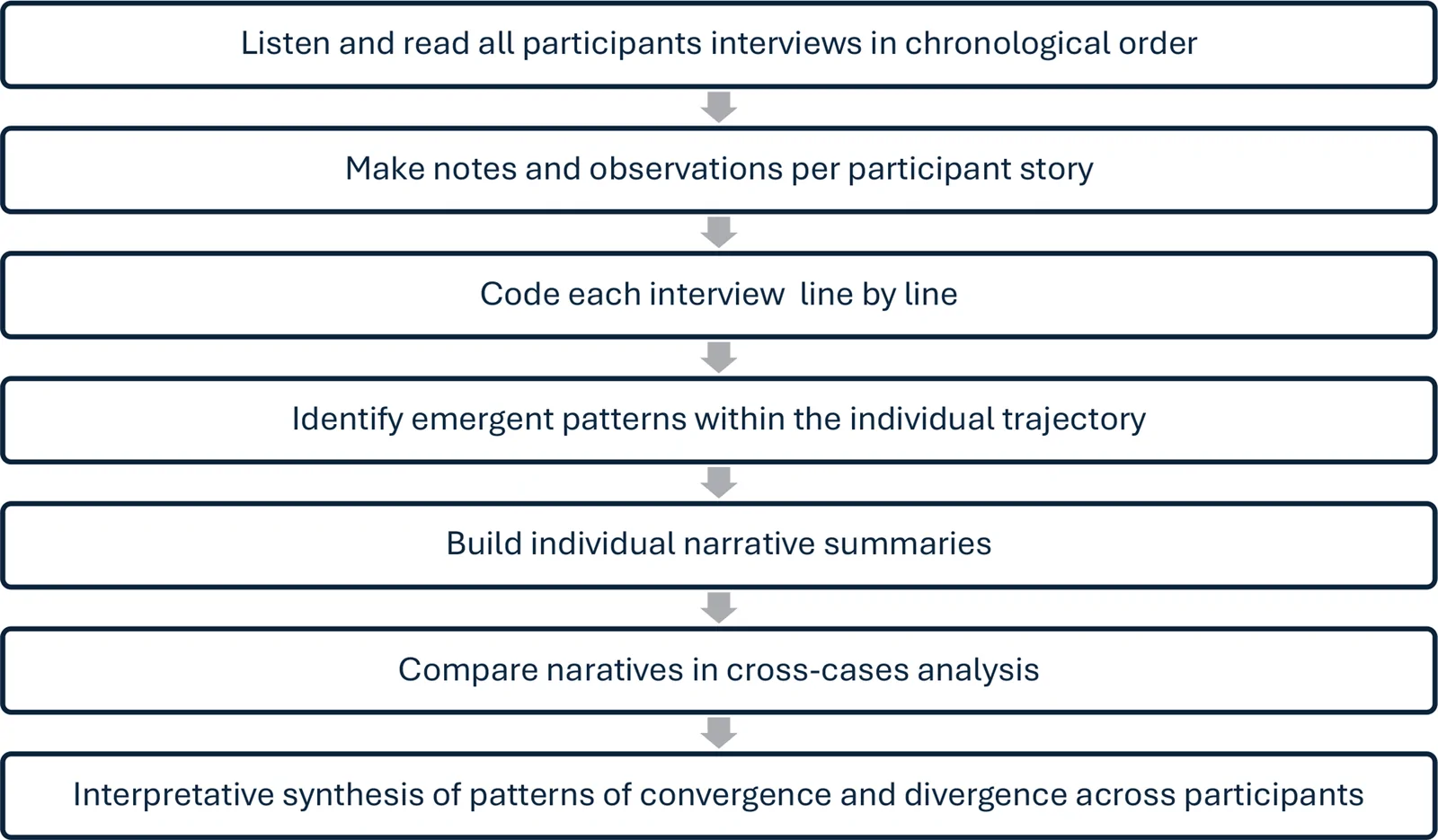
Schematic representation of the data analysis structure.

### Reflexivity statement

The lead researcher is a Brazilian woman sports physiotherapist with extensive experience in elite and high-performance sport and a postdoctoral researcher with substantial expertise in qualitative research. Her professional background, being a clinician who worked with athletes from different cultures and backgrounds, facilitated communication with participants and supported a contextualised understanding of the athletes’ experiences, including sensitivity to their emotional burden.^33^ We acknowledge that cultural context shapes how athletes experience and narrate their journeys; and the lead researcher’s Brazilian background and international experience in elite sport informed her sensitivity to these cultural nuances. At the same time, we acknowledge that this positionality may have shaped interpretations of athletes’ experiences, particularly regarding rehabilitation practices and sport culture. The author team comprises professionals from diverse disciplines, nationalities and professional backgrounds. The team discussions served as an opportunity to critically examine emerging interpretations, consider alternative understandings of the data and refine the developing structure. The analysis was strengthened by challenging assumptions and ensuring that interpretations remained grounded in participants’ accounts while acknowledging the researchers’ different disciplinary and professional perspectives.

### Equity, diversity and inclusion statement

This study focuses on a diverse sample of athletes, two men and three women, in various moments of their career, from North America (USA), South America (Brazil) and Europe (Italy, Malta and the Netherlands) playing team and individual sports within different resources and infrastructures. The author group is composed of five male physicians, two sports physiotherapists and two sports scientists. The authors’ group was led by a woman middle career researcher from Brazil, supported by seven men from the Netherlands (3), France (1), Italy (1) USA (1), the UK (1) and a woman from Australia.

## Results

Five athletes ranging from 19 to 42 years of age, representing various sports (volleyball, tennis, football, judo and wrestling) and countries (Italy, Brazil, the USA, the Netherlands and Malta), underwent 31 interviews conducted from April 2023 to September 2024. A detailed description of the interview dates and duration is presented in the online supplemental file 2. Box 1 provides a summary of the athletes’ background and context. This contextual detail helps situate each journey within its specific sporting, cultural, and organisational environment. The detailed individual narratives are available in the online supplemental file 3.

Box 1 **Summary of the athletes’ characteristics, background and context**

**C, 42 years, volleyball player, Anterior Cruciate Ligament (ACL) tear, first interview 10 days after surgery. Total of six interviews.**

She had many career accomplishments, already thought of retiring in the upcoming years, and had never had any severe injury. She has been with the same club for many years and trusts the healthcare team, with whom she has an established relationship, an easy diagnosis process and planning and surgery procedure. Supported by family, PT and friends. Motivated for the Olympics but concerned about the potential impact of the injury, as she needs to be back playing to be noticed by the national coach to be selected. She sees the injury as an opportunity to focus on her well-being and on her life beyond sports.

**D, 19 years, tennis player, fifth metacarpal fracture, first interview 25 days after surgery. Total of four interviews.**

His career is starting to further develop, and he is reaching some of his career goals. He was just coming back from a long-term overuse injury when the injury happened. He was in another country, and he had to go through surgery abroad, but had a good network in his own country that facilitated communication with foreign doctors and guaranteed the quality of the healthcare. There is an uncertain prognosis and questions whether the athlete will be able to return to tennis. He has a good support system from his parents and coach. Feels frustrated with the pain, the lack of activity and not being able to play.

**J, 19 years, football player, ACL tear, first interview 3 days after surgery. Total of seven interviews.**

He went through surgery in the previous season due to another injury and feels that the previous injury helped him to better deal with this one. He is playing his first year in the first league team in his country, with previous experience playing youth football in England. He hopes for new career prospects. Has a good network for healthcare, trusts the doctor and the club physio, who helped with the previous injury. He has good social support and other professional activities. He wants to succeed in football, but questions if he is getting old and wishes for new career opportunities.

**A, 29 years, wrestler, ACL tear, first interview 8 days after surgery. Total of seven interviews.**

After the ACL rupture, she tried to continue to compete but opted for surgery. She is recently married, clearly stating the importance of her religious faith in dealing with the injury. The injury is challenging mentally for her despite the good healthcare and established relationship with health professionals in a training centre. She has some thoughts about retiring, but doesn’t want to think about it before she recovers completely. While she tries to be positive and has the right motivation for the Olympic Games, she acknowledges the impact of the injury on her sports performance.

**E, 26 years, Judo athlete, hamstring tendon rupture, first interview 17 days after surgery. Total of seven interviews.**

She had career achievements at an early age, with a promising future, but has a long history of multiple injuries with previous surgeries in different parts of her body. The new injury makes her doubt her future in sports. Her previous injuries are a big toll for her to carry and impact her expectations for rehabilitation and future career prospects. She did the surgery abroad, lives in a training centre, and feels supported by the coach, parents, friends and a mental coach. She balances the potential future prospects, the toll of previous injuries, the fear of a new injury and her love for the sport. She considers that her injury is healed and resolved, but she decided to stop her sports career during the current study, which is a tough decision, as she feels she is failing as she renounces her dreams.

The cross-case analysis revealed three overarching journey phases, each characterised by recurring factors, emotions, and circumstances, which often overlapped and did not follow a strictly linear sequence. Despite these shared themes, the athletes’ narratives demonstrated substantial heterogeneity. For example, social support was perceived as either beneficial or detrimental, and in some instances, its influence shifted over time for the same individual.

Tables 1–3 present illustrative quotations for each phase, thereby supporting the key concepts presented in the text in **bold**, participants are identified by a letter followed by the number of the interview, and figure 2 provides a graphical representation of the athletes’ trajectories, illustrating the fluctuations across the three phases and their associated key concepts.

**Table 1:** Key concepts and quotes related to ‘the rough start’

Concepts	Quotes
**Lost identity**	*All I want to do is play football; my personality is built on football. (J 02)* *You grow in a way, and you think that your life is Judo, that Judo is your life. Your goal in life is to go to the Olympic Games. You work and work and work. (E 01)*
**Uncertainties**	*I was pretty scared in a way because they literally told me, ‘We are not sure if you can play tennis again pain-free.’ So that’s kind of a hard message to swallow because, yeah, tennis is my life. (D 01)* *I didn't expect to go through this now. Because I'm 42 years old. I had already planned for another two to three years of volleyball. So, this year, I had more or less programmed what I was going to do. I was going to finish the season, I was going to the national team, go to the pre-Olympics, play one more season and try to get a place to go to Paris (Olympic Games) next year, which would be my big goals. Later, I would retire. And out of nowhere, an injury like this happens. An injury that is not simple, it’s a long treatment, it’s a treatment that we will need a lot of patience, a lot of resilience and everything. (C 01)*
**Sad and frustrated**	*It’s not easy, it’s too high, too low. There are days when I go to sleep and, suddenly, out of nowhere, I start crying. Then, out of nowhere, okay, I stop crying, and the next day I'm fine. So, I get more emotional… There are better days and worse days, but I'm trying to take it the best way possible. (C 01)* *I have cried every day since the fifth of March. I had the injury in the competition on the fifth of March. I have cried every day. (E 01)*
**Injury timing**	*It was like a journey for me. With the previous injuries, I was still young, so I still had time. But with this one, I had everything in place to prove myself this year. Next year, try to go abroad. So it’s a bit hard to take it, because I'm getting older now as well. And I had lots of opportunities this year as well with the national team. (J 01)* *But it’s really, really hard now for me, to qualify for the Olympic Games because I have a short time when I come back to qualify myself. (E 01)*
**Feeling guilty**	*I take care of myself a lot, I take care of my diet, I take care of my sleep, I take care of my rest, I do all I can. And then, when the injury happened, I kept asking myself, but what did I do wrong? (C 01)* *So I think and overthink, if I have done something wrong or if I could train more or if I could sleep a little more, anything. (E 01)*
**Navigating the healthcare system**	*It was really challenging. You know, some doctors, their English wasn't really good. Pretty hard to understand sometimes. And also, I never experienced something like this. And I don't know any of the doctors. I don't know the hospital. I don't know the place. (D 01)* It was easy. The good thing about all is that this happened here (name of club). The (name of doctor) accompanies me; he has been taking care of my knee for years, so he already knows everything about me. He knows what my knees are like. And he’s a f**ing competent doctor, you know? So I super trust him. And he operated with (name of doctor), who is also another great doctor. (C 01)
**Coping strategies**	*I'm just taking it day by day at the moment. I'm not trying to think about what’s gonna happen in the next year. I'm just focusing on the rehab and focusing on getting back as soon as possible.* *(J 01)* *I'm always believing that the hard work pays off. So I try just to take it day by day. Keep believing that if I keep working hard, the good results will come. That’s all you can do. (D 01)*

**Figure 2:**
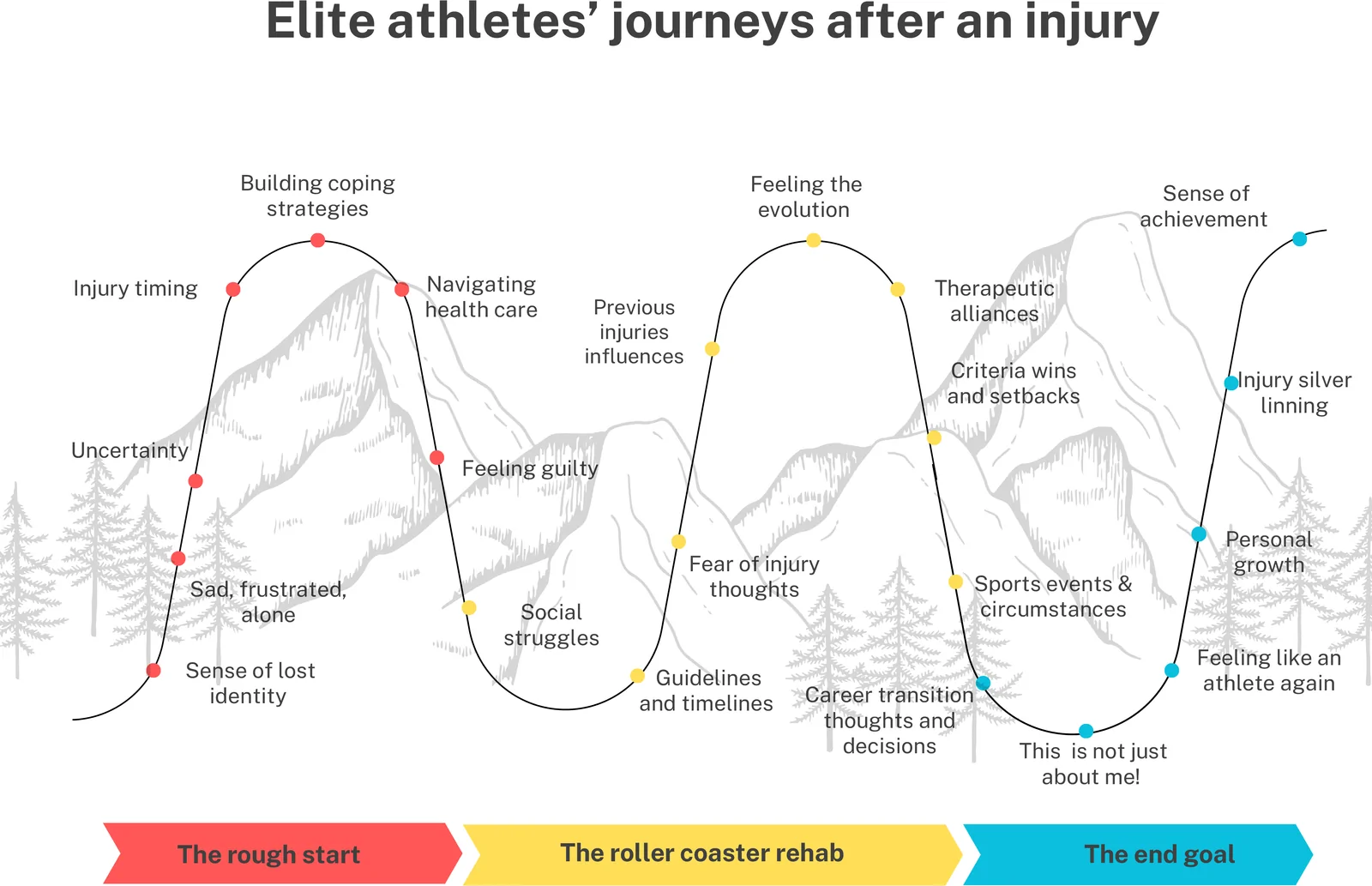
The main outcomes of the analysis revealed three phases of the athlete’s journey: ‘the rough start,’ ‘the roller-coaster rehab’ and ‘the end goal’ and their associated key concepts, illustrating fluctuations and diversity of experiences across different athletes’ journeys.

**Table 2:** Key concepts and quotes related to ‘the roller coaster rehab’

Concepts	Quotes
**Feeling the evolution**	*The recovery is going well, and I can do everything. I can do all: squat, deadlift, clean, and judo. My leg is okay. It’s not the same. Not as strong as the other, but strong anyway, I'm still doing all the specific exercises. (E 04)* *And of course, if you look at a longer period of time,* like 6 weeks *ago, of course, the movement improved a lot (…) the movement is getting better, has got better, but especially pain-wise, I would say. (D 02)*
**Criteria wins and setbacks**	*These numbers (isokinetic test values) are created based on a lot of past patients (…), it’s general, it’s a guideline. It’s accurate for most people. And I wanted to be in the majority. And I had to make it, I felt like I needed to work up because I'm a goal seeker. You put something in front of me, and then I go chase it. So these numbers helped me. My impression was that quantifying your work by putting numbers to your progress is very encouraging…and discouraging. I just didn't want to be outside of the average in either direction. (A 07)* *I was bending my knee yesterday, and it was 115. Then today, almost 120. So, it’s a victory for me, and we celebrate. So, it’s a celebration; they are small celebrations. Today I'm already walking better, it’s already a celebration for me. (C 02)*
**Previous injury experiences**	*Obviously, being injured for like every season, like big injuries for every season, is quite hard, but with me getting injured in previous years, for this one, it has helped me a lot, but obviously, I'm more frustrated and angry that I'm getting these injuries over and over. (J 01)* *Every surgery that I have done has been worse and worse each time. And it was worse each time to restart judo. I was more patient. I was more, I don't know how to say, but every time I need more recovery. Now I think my mind is like, ‘What’s the next surgery if I start to do that again?’ What’s the next thing that I have to work on in my body? (E 04)*
**Guidelines and targets**	*The guidelines mean a lot to me because it’s a step that you sort of reach, and then you build on it. So that’s a sign of improvement. It’s a sign of progression. (J 02)* *I got stuck at 90 degrees for flexion. For weeks. And I was really sad about it because I was trying so hard, but I couldn't, I couldn't pull my leg, I couldn't pull it towards me anymore. It just wouldn't let me. And so I got frustrated, and that was maybe two and a half or 3 weeks of just trying to break 90 degrees. (A 02)*
**Communication challenges**	*The first few weeks I was a bit stressed. And, to be honest, now I'm just gonna sort of play it along day by day. I support the physio of the club, that’s the one I want to work with, to be honest. Because the physio of the national team, I don't really like. It’s not because I don't like him, but I don't like his ideas, you know, how he works. Then, actually, the team is the national team, and also you can't really fight with them or say: ‘Oh, I'm not going to do this’, it’s a bit tricky. (J 02)* *The trainer is saying: ‘Go with the coach’ and the coach says: ‘That’s not realistic’. And so I'm in the middle of these, I'm playing mediator between my medical and my sports people. So their expertise is in opposite things. And I don't think the coach fully understands where I'm at physically, and leaves it up to me in a very ambiguous way. And I don't feel like I have enough guidance right now to know where my re-entry needs to be to keep me on track for the Olympic trials. So I can't make both happy. (A 04)*
**Sports events and circumstances**	*I didn't play, I couldn't go to training, I couldn't be part of the team. It was a mix of a lot of things that were happening in the season. So, I had no desire to be there. I felt a little frustrated; the frustration of not playing. And in the finals, that was very clear. The frustration of not being able to enter the court, in the finals. It’s frustrating to feel like that. It is impotence, you know? From the national team as well, I confess that when the call-ups started to come out, I felt very bad for not being there, not to be part of it - it was too painful. (C02)* *In my category, another girl is competing for the Olympic path, and she is younger; the national team prefers her. (E 03)*
**Fear of injury thoughts**	*I'm scared of not being prepared to take the weight off my leg when something’s coming to my leg. So twist is the scariest motion because it makes me nervous. (A 03)* *Obviously, it doesn't cross my mind that I'm gonna get injured. But obviously, sometimes you think about it. When you see, maybe another guy just got injured. You know what I mean? So this sort of stuff takes you back to when you were injured. But I'm not, I'm not fussed about, or I'm not afraid about getting injured or this sort of stuff. No, no. (J 05)*
**Therapeutic alliances and trust in health professionals**	*He is a physio at the club. So I went to (name of the club))last year. So he’s the same physio that we had last year. I already did a recovery period with him as well last year. I had that operation, I did 2 months with him, so we're quite close. So in terms of the rehab, I'm quite not looking forward, but I'm quite certain that’s going to be good because I know I'm in good hands. (J 02)* *From the beginning, my physiotherapist (name))was the main support. I say that if it weren't for her, maybe I wouldn't be going through this process in the way I'm going through, calm like that. Of course, it’s difficult, there are worse days, there are better days, but she gave me such a welcome and support at the time. (C 03)*
**Social support and distancing**	*I have been abandoned by my coaches and even by my physiotherapist. I had done physiotherapy, but he didn't stay by my side. I was alone in this process. I was alone to do the exercises. I was alone. (E 04)* *My parents, they support me every day and in everything I do. Second of all, my coaches. We always kept in touch, even in the difficult times and also the doctors and the physio. Yes, they're really supporting and helping me with what they can. So these are the most important people. (D 02)*

**Table 3:** Key concepts and quotes related to ‘the end goal’

Concepts	Quotes
**Feeling of accomplishment**	*Given all the conditions, all the situations I've been through, this is pretty, this is a lot. I just got done with knee surgery, cleared and went straight into Olympic trials. Not how I wanted it to go, but I was like, I'm impressed with this, I'm proud of myself. I'm proud of myself that I even got through the surgery optimistically. And at least the tournament itself motivated me to get my health back. (A 07)* *But an injury can also be like good for some things, you know?! Because you feel, I would say, you feel like, stronger when you come back from an injury. And also, when you win a few good matches, it feels even better after an injury because you know what took you to get there, and the feeling when you win after an injury is even better than the feeling when you're playing for like a year straight. (D 03)*
**Feeling like an athlete**	*But now I'm feeling a little bit like a player and not like a person who is rehabilitating, you know? (C 04)* *The knee is reacting very well, thanks to God. It’s, it’s good now. It feels more like football. Now, I feel more like an athlete, like I'm, I'm getting back. (J 04)*
**Personal growth**	*I think mentally, I think I changed a bit on how I think about life. I think I changed for the better, you know, I grew up, I grew from it. (J 07)* *As a human being, I think that’s it. I grew a lot in this phase. I can look more in general, at a bigger picture. Not only that volleyball athlete thing, but in a bigger environment. So I think I gained a lot of perspective from it. (C 06)*
**Silver linings of the injury**	*When you have an injury, and you know that you're not going to have a match for a few months, you can practice things. Like change things in your game, which you maybe cannot do when you're playing tournaments every week. (…) So in a way, it also helps your game because I'm a bit of a conservative player (…) I started to practice a lot of attacks, like the attacking style (…). It’s going pretty well. So, there are also new additions and things in my game that I added, so I would say I'm already pretty close to the level I was before the injury. (D 03)* *There are also other things that sometimes we are so focused and so immersed in that world of sports or high performance of what you want, that you end up not seeing things from the outside. I got much closer to my nephews, my family, who for me was very important. I had time to go home, I had time to travel, I did spend a lot of time with my mother. So in this whole process, there were some very good things (C 05)*
**Career transition thoughts and decisions**	*I think the injury gave me more of a perspective on, I won't be doing this forever. And what other things would I like my life to look like? And it humbled me. And I know that I can probably make a career change at some point. (A 05)* *So the leg it’s ready, but me: I am not. I remember that when my friend said to me that, in his opinion, I have done the wrong decision because the time for the Olympic qualification is not over. He said to me, the time is not finished. So you have to try it until the time is finished. And I answered, I thought about 2 min. And then I said to him: ‘The Olympic qualification time is not finished, but my time is finished’. (…) For the first time in my whole life, I prefer to save my body instead of saying I will risk everything to maybe go to the Olympic Games. (E 05)*
**It’s not just about me!**	*Because people are very anxious for my return. This, sometimes makes me a little more insecure. I need to put my head down, because there’s a lot. People are very anxious. They want me to come back, they want me to come back. But more from the outside, they're fans, they're people who know me, they're reporters. I'm getting ready for it. I want to come back when I'm good, but I feel like there’s going to be this pressure when I'm in there, when I'm available there to play. He (coach) will have the pressure of having to put me to play, and he doesn't want that, because the group is good, the group is playing well. (C 05)* *I started training with the team (…). Then I had a chat with the coach, and with the club, I signed an extension of the contract. I signed another 2 years with the club, but obviously with the aim to go on loan this year and to gain game time and confidence back. So, for this year, I signed with a lower division club. (…) Obviously, there’s still a couple of things I need to improve in terms of sharpness and speed, and this sort of things comes from games rather than gym strength, you know?! (J 07)*

### The rough start: the struggle is real

In the first interviews, athletes described a **sense of lost identity**, the memories of the injury incident and the **uncertainties** about their future and the long recovery left them **sad, frustrated** and depressed. It was common to hear expressions like “my world fell apart” C, or “I have cried every day” E. The **timing** of the injury was often mentioned, considering the proximity of the Olympic Games, their career and their future plans. Many times, athletes described **feeling guilty** or asking, ‘Why me?’.

Some athletes also had to **navigate the healthcare system** in search of experts and a clear prognosis. Athletes integrated into a system with established relationships with health professionals were more easily assisted. Two athletes had to undergo their surgeries outside of their country, facing uncertainty and a lack of social support due to being away from their usual environment. The athletes often mentioned the need to create their **coping strategies**, such as maintaining a positive mindset, relying on the support of family, friends and health professionals, or focusing on activities outside their sport life.

### The roller coaster rehab: improvements, setbacks, miscommunications and little wins

During the follow-up interviews, athletes described moving through different phases of rehabilitation and **feeling the evolution**. Improvements were identified through clinical and objective criteria, such as pain reduction, increased range of motion, regained function in sport-specific tasks, and subjective signs of progress, including everyday activities like helping a friend move or taking long walks while on vacation. There was a mix of **wins and setbacks based on various criteria**. Improvements provided tangible markers that motivated athletes. When progress was delayed or expected milestones were not met, athletes reported feelings of frustration, which in turn influenced their motivation. Injuries with well-established guidelines, such as Anterior Cruciate Ligament (ACL) reconstructions, were more structured based on clinical goals and an expected timeline, while injuries with no established guidelines relied mostly on individual outcomes and time-based goals.

Athletes frequently reflected on **previous injury experiences** to make sense of their current situation. Some athletes with prior injuries felt knowledgeable and familiar with the process based on their past recoveries, while others carried the emotional burden of setbacks related to previous RTS. The **fear of a new injury and reinjury** was mentioned a few times, sometimes also influenced by their previous injuries and those of other athletes.

**Sports events and circumstances**, such as national team competitions, upcoming career opportunities, the rise of a new athlete and the signing of a replacement contract, were often discussed during the interviews. The athletes commonly described moments of more emotional difficulty with these events affecting their motivation in both positive and negative ways.

The **communication challenges** were also frequently mentioned by the athletes. Some athletes struggled to interpret and reconcile differing advice from various stakeholders, such as surgeons, coaches and physiotherapists, also from different organisations such as the national team and the club. They often found themselves acting as mediators between professionals and felt frustrated when conflicts arose. In contrast, athletes supported by a more coordinated and communicative team experienced fewer issues and greater clarity. The communication issues often led to misaligned expectations and emotional strain.

The value of the **therapeutic alliance, familiarity, and trust in health professionals** made athletes more confident and engaged in the process. The support of family and friends also facilitated their journey. However, many athletes also spoke about the emotional toll of seeing teammates compete or watching key matches while being injured, and their need to create **distance** by avoiding watching competitions, social media or even travelling to disconnect from the sport context.

### The end goal: owning the process and gaining new perspectives

In the last interviews, there were a few moments described by the athletes with a **sense of accomplishment** after going through a recovery journey. They perceived the late stages of rehabilitation made them rescue their athletic identity and **feel like athletes again**, sometimes because of being back training with teammates or including sports-specific activities and demands in their routines.

There was a shift in the meaning of their RTS. Whereas early conversations focused heavily on returning to their previous level of performance, later reflections revealed a broader perspective and **personal growth**. Several athletes described the injury as a learning experience leading to unexpected positive outcomes and lessons. Some **silver linings** of the injury were appreciation for life beyond their athletic, personal growth, development of mental resilience, becoming better players, recognising the importance of long-term health, and gaining clarity on the finite nature of their athletic careers. One athlete even referred to it as “the best thing that has happened in my life recently.”

As the athletes reflected on their journey, they perceived a change in priorities. All participants considered the time away from sports as an opportunity to explore an identity beyond sport. For some athletes, this new perspective made them think about **career transitions**. Two athletes decided to stop their athletic careers. They acknowledged that the injury ignited the process but did not consider the injury the reason for finishing their career. They felt physically rehabilitated and had the option to compete again. One athlete struggled with fear of regretting and felt unsupported by health professionals when she tried to discuss her reflections about not returning to sports, while the other athlete was satisfied with her decision and inspired by other athletes in her circle who had gone through a career transition before. Even in cases where individuals did not return to performance and ended their careers, the focus was not on the injury itself and the individual narratives, experiences and contextual factors influenced the process.

Athletes also described how returning to competition or performance **was not just about them**, influenced by factors beyond their physical or mental readiness, especially late in the process. They mentioned external elements such as team dynamics, contract opportunities, competition schedules, media pressure and sponsor expectations. Meeting the RTS criteria did not necessarily mean returning to the team or competition, as the competition timing or team situation sometimes did not allow for a full return to performance shaped by more than just their physical recovery.

## Discussion

This longitudinal qualitative study in five athletes with an RTS prognosis of at least 4 months highlights how RTS is a highly individual journey shaped by identity, uncertainty, communication, social context and evolving career perspectives. Although athletes’ experiences could be broadly organised into shared phases, substantial variability was evident both between athletes and within individuals over time.

### The individual, dynamic and non-linear process

This study reinforces that recovery from severe sports injury is not a linear or uniform process but a highly individual experience that evolves over time and context. Although common patterns were observed across athletes, their needs, priorities and responses differed, not only between individuals but also within the same athlete at different moments of recovery.^34^ The injury impact for athletes is aligned with the concept of biographical disruption from medical sociology which describes a disruption of how people understand themselves.^35^ This finding aligns with previous qualitative literature describing injury as a disruptive event that challenges identity, goals and assumptions about the future.^13 36 37^

Existing evidence often describes recovery using broadly defined phases such as career threat, identity loss, adaptation and reflection.^38–40^ While such results showed how athletes perceive their injury recovery journey, our study illustrates that athletes may move back and forth between perspectives, experience uncertainty even late in rehabilitation or redefine success in ways that are not captured by standard models.

From a clinical perspective, this challenges the widespread use of highly prescriptive rehabilitation pathways and RTS frameworks.^41^ Protocols and guidelines can provide structure and safety but when applied rigidly, they may fail to acknowledge the individuality of the recovery process.^14 42^ The athletes’ perspectives showed that the most meaningful guide for rehabilitation progression is not the protocol itself, but how the athlete is responding physically, psychologically and socially at a given moment. Clinicians should therefore use protocols as references rather than rules, adapting them in response to the athlete’s evolving needs and context.

### Therapeutic alliance, communication and tailored goal setting

The influence of health professionals emerged as a central element shaping athletes’ rehabilitation experiences. Athletes who described strong, continuous therapeutic relationships reported greater trust, engagement and confidence throughout recovery, which was previously reported by the literature.^6 13 43^ In contrast, fragmented care, inconsistent messaging or poor communication created confusion and frustration. Particularly when athletes moved between organisational settings such as clubs and national teams, showing the importance of communication and clear handovers.^44^

These findings are well supported by existing evidence highlighting the role of therapeutic alliance, expectation management and shared goal setting in rehabilitation outcomes.^45 46^ However, this study adds nuance by showing how goal-oriented processes can become counterproductive when goals are overly rigid or insufficiently tailored. While criteria-based milestones motivated many athletes, failing to meet predefined targets within expected timelines often led to self-doubt and questioning of the rehabilitation process.

Clinically, this highlights the importance of transparent, ongoing communication. When progress does not follow expected timelines, reframing goals, explaining uncertainty and contextualising delays can help maintain motivation and trust. It is known that supportive environments that reinforce competence, autonomy and relatedness are beneficial for RTS outcomes.^47^ Rather than focusing solely on ‘meeting criteria’, clinicians may support athletes more effectively by revisiting the goals regularly together and ensuring they remain realistic, meaningful and aligned with the athlete’s current situation. This reinforces the value of engagement, adaptive communication, empowerment and shared decision-making as core components of athlete-centred care.^4 7 8 48^

### RTS as a contextual and system-dependent process

This study highlights that RTS is shaped not only by physical readiness or test outcomes, but by the broader sporting context in which the athlete is embedded.^12^ Differences in injury type, sport-specific demands and available evidence influenced the structure and predictability of the rehabilitation process. The three athletes recovering from ACL injuries often followed clearly defined pathways, reflecting the strong evidence base in this area. In contrast, athletes with less common or less researched injuries faced greater uncertainty, fewer reference points and less structured guidance.

Beyond injury type, sport-specific and organisational factors strongly influenced RTS experiences. Team dynamics, competition schedules, selection policies, contracts and replacement decisions all shaped opportunities for reintegration, reinforcing the idea of the RTS complexity with multiple levels and influencing factors beyond athlete-related aspects.^49^ Athletes described needing cooperation from teammates, access to match play or negotiations with clubs to make RTS possible. While the complex nature of the RTS was previously described, our study described a diversity of factors which often lay outside the clinician’s direct control but had a clear impact on motivation, confidence and perceived readiness.

Existing RTS models largely emphasise physical and psychological readiness metrics,^5 50^ with less attention to these contextual realities.^9^ Our findings in severe injuries (over 4-month RTS prognosis) suggest that RTS decision-making should extend beyond individual criteria to include an understanding of the athlete’s sporting environment. Such a concept is theoretically suggested by the Strategic Assessment of Risk and Risk Tolerance framework,^2 3^ but in practice, physical and clinical criteria overpowered the contextual and risk-modifier factors. Clinically, this means recognising that successful RTS planning requires awareness of team structures, timing within the season and external pressures that may either facilitate or hinder reintegration.

### Career transitions and decision-making beyond RTS

An important contribution of this study is the documentation of athletes who chose not to return to competitive sport after a severe injury. These decisions were not necessarily framed as failures or a result of career-ending injuries, but as outcomes of reflection, changing priorities and reassessment of long-term health and life goals. This aligns with literature describing injury as a moment that can prompt broader identity and career reconsideration.^51–53^

Despite this, clinical practice often remains heavily focused on restoring function and achieving RTS, with limited attention given to supporting athletes who are uncertain about returning or considering alternative pathways. Some athletes in this study indicated a lack of guidance and support during decision-making about their future, highlighting a gap in current clinical education and practice.

Health professionals are well-positioned to support these transitions by creating space for open discussion, validating uncertainty and helping athletes explore options.^52 53^ There is a need to prepare medical, performance and coaching staff to support career transitions, helping athletes to navigate different life phases and supporting their athletic and non-athletic identity.^51^ While sports psychology as a field developed a broader understanding of career transitions in sports,^54^ most of the other health professionals are not fully equipped with the knowledge and skills to support athletes. Supporting career transition does not mean discouraging RTS, but recognising that recovery may lead to different outcomes for different athletes. Actions and interventions supporting athletes after their sports career should be more explored.^55^ Clinically, this calls for a broader definition of successful rehabilitation, one that includes informed decision-making, long-term well-being and alignment with the athlete’s values.

### Growth, reframing and shifting social support needs

Several athletes described positive changes emerging from the injury experience, including increased resilience, improved self-awareness and a broader life perspective. Such ‘silver linings’ have been reported previously.^38 56 57^ By following the athletes longitudinally, our findings show that growth was not automatic and often coexisted with ongoing challenges. Reframing the injury experience was influenced by social context, current and future career opportunities, quality of support and interactions with health professionals.^58^ This dynamic and context-dependent nature of psychological adaptation aligns with previous sport injury literature highlighting the growth after an injury and the role of ongoing cognitive appraisal processes, evolving social support, rehabilitation environments and relational dynamics in shaping athletes’ responses to injury.^59–62^

Contextual and social influences were dynamic rather than static. Resource availability extended beyond access to care to include familiarity with healthcare systems, trust in professionals and quality of therapeutic relationships. Similarly, social support needs shifted over time. Early in recovery, athletes often relied more on family and non-sporting contacts due to physical and emotional distance from their teams. Later, teammates played a key role in restoring belonging and athletic identity. Therefore, while social support and healthcare systems are often mentioned as success factors for RTS,^6 17^ the needs and expectations change over time during the recovery process. These findings can also be interpreted through the Integrated Model of Psychological Response to Sport Injury,^63 64^ which emphasises the interaction between personal factors and situational factors such as social support in shaping cognitive appraisals, emotional responses and behavioural outcomes. The athletes’ narratives in this study illustrate how these factors jointly influenced their interpretations of injury, coping strategies and emotional adjustment.

Clinically, this highlights the importance of recognising when and how support needs change during recovery. Encouraging reflection, maintaining connection with the sporting environment and supporting social engagement may facilitate adaptation and psychological well-being, which reflects on physical outcomes. Understanding these shifts can help clinicians better time their interventions and strengthen the therapeutic alliance.

### Methodological considerations

A key strength of this study in severe injuries (prognosis of>4 months RTS) lies in its longitudinal qualitative design. Repeated interviews made it possible to explore how psychological, social and contextual factors shifted over time, even within the same athlete. However, recruiting participants was challenging due to the commitment required for multiple interviews and the timing for inclusion. Nonetheless, we included five athletes from five different countries, sports and injury types, capturing a diverse range of experiences across both well-resourced and less structured sport systems. While this diverse sample enriches contextual understanding, we acknowledge that including participants with varied backgrounds could have reduced the depth of exploration within any single context. However, we believe that the diversity of contexts (different sports, countries, career stages and healthcare systems) helped us to understand how the broader elite sport system, in all its contextual variability, shapes athletes’ RTS journeys. We did not aim to conduct a formal cross-cultural comparison or to test cultural differences systematically.

Some athletes were recruited through professional contacts, and although anonymity was guaranteed, this prior connection could have affected their openness. Additionally, we acknowledge the burden placed on participants, both in terms of time and emotional investment, which might have impacted the level of engagement.^33^ Despite the high demand, all participants maintained contact with the main researcher, showing availability for new interviews, without any drop-out. Quality was supported through sustained engagement with participants’ narratives over time and through an iterative interpretive process. Analysis involved repeated movement between individual cases, longitudinal trajectories and cross-case patterns, alongside regular discussions among the research team to challenge interpretations and consider alternative understandings. Reflexive attention was also given to how researchers’ backgrounds and experiences informed the analytic process.

Participant well-being was actively monitored during interviews. The lead researcher, drawing on her clinical background, was attentive to signs of emotional distress. Participants were reminded at each interview of their right to pause, skip questions or withdraw. Some of the participants were supported by mental health professionals while others considered no need of additional support. In some cases, especially after returning to sport, athletes had tight schedules and there were long gaps between initial contact and interview completion. This may have impacted the continuity and detail in how their experiences were described. To support transparency and reflexivity, a structured analytical process was followed in which each participant’s case was examined individually and in chronological order before cross-case comparison was undertaken. This approach preserved idiographic depth and allowed the researcher to remain attentive to within-case change over time. It is also possible that athletes who agreed to repeated interviews differed from those who declined (e.g. in openness or available time), which may have shaped the range of experiences captured. Participants had access to their interview transcripts. However, we chose not to incorporate formal member reflection or collaborative analysis processes, as repeated engagement with the large volume of longitudinal data would have required a substantial commitment from participants and may have increased participant burden. Although only one researcher led the analysis, a full audit trail and regular peer debriefing supported the credibility of the process.

## Conclusions

This study provides an in-depth exploration of the psychological, social and contextual factors that influence RTS across a diverse range of scenarios involving different sports, injury types and healthcare systems. This reinforces the importance of acknowledging that athletes are likely to be changed by their injury experience in different and individual ways. Shifts in how success and recovery were defined extended beyond physical performance and were shaped by personal circumstances, contextual pressures and clinical interactions. These insights highlight the importance of walking alongside the athlete throughout their journey, recognising the evolving interplay of personal, social and structural factors. To deliver individualised and context-sensitive care, clinicians and support teams must continuously adapt to the changing aspects of each athlete’s experience.

## Supplementary material

10.1136/bmjsem-2026-003320 online supplemental file 1

10.1136/bmjsem-2026-003320 online supplemental file 2

10.1136/bmjsem-2026-003320 online supplemental file 3

## Data Availability

All data relevant to the study are included in the article or uploaded as supplementary information.
