# Supplementary material for: From ‘My world fell apart’ to ‘The best thing that happened in my life’: elite athletes’ longitudinal journeys after an injury
Source: BMJ Open Sport Exerc Med. 2026 Sep 25;12(3):e003320. doi: 10.1136/bmjsem-2026-003320 (PMC13630066; doi:10.1136/bmjsem-2026-003320)
Supplement: Supplementary file 1 [file bmjsem-12-3-s001.docx]

**Individual narratives**

**A, 29 y, wrestler, ACL tear, 1^st^ interview 8 days after surgery. Total of 7 interviews**

**Interview 1 – Injury, Surgery, and Heartbreak**

At 29, newly married and still chasing Olympic dreams, A. tore her ACL during a World Cup. She tried to push through and compete for months, but her wrestling became defensive, her body never fully right. Surgery became her best option. After surgery, she felt lonely, being away from training and teammates, and uncertain about the future. At times, she wondered if she should quit wrestling altogether and start a new career. Yet faith, her husband, her trusted physiotherapist, and friends kept her grounded. Above all, she clung to her belief that she would regret stopping now.

**Interview 2 – Early Progress, Lingering Fear**

Eight weeks post-op, A. was encouraged: she had regained knee motion, trusted her surgeon’s work, and was ahead of schedule. Still, she feared retearing and often overprotected her knee. She got stuck on range-of-motion gain; she questioned her responsibility for the setback, but regained motivation when clinical improvement came.

She balanced her uncertainty about the Olympics with small, daily goals, while keeping a distance from competitions that reminded her of what she was missing. Hobbies, faith, and support helped her cope, but she admitted she was still living with doubt about the future.

**Interview 3 – Confidence and Hope Returning**

By four months, A. was gaining motivation and confidence. Her function was constantly improving; she was running on the treadmill, helping friends move, and beginning to think about sport-specific training again. She admitted feeling nervous but also excited about returning to the mat. She recognised the injury had given her something she’d never had before — the space to enjoy life outside wrestling. Mentally, she felt stronger, anchored by faith, and motivated to chase both short-term goals (Olympic qualification) and long-term ones (a career beyond sport).

**Interview 4 – First Return to the Mat, Frustration with Communication**

Stepping back on the mat for the first time was thrilling but also overwhelming. Coaches pushed her to wrestle, while medical staff warned she wasn’t balanced or ready. Stuck between contradictory advice, A. often felt she was the only one making critical decisions. Functionally, she felt strong and confident, but communication gaps left her frustrated. Despite this, she remained driven by dreams of the Olympics, even as she struggled with mixed messages.

**Interview 5 – Setbacks and Realignment**

At 25 weeks, testing revealed her quadriceps strength was far below expectations, bringing guilt and self-doubt. The communication between professionals improved with an aligned approach. With clearer communication and shared planning, A. felt more supported, even if her return had to be delayed. The timing of her return was also challenging, given the competition calendar and her opportunity to compete before the Olympic trials. She also leaned more into thoughts about life after wrestling.

**Interview 6 – Confidence with Perspective**

As her team began working more effectively together, A. regained confidence. A psychologist was supporting her to learn to “trust my body and get into sport confidently. She managed anxiety and accepted that she would not be fully competition-ready before Olympic qualification. Still, she felt stable, encouraged, and proud of the progress she had made. For the first time, she seemed at peace, balancing her Olympic hopes with the reality of transition toward a new career.**Interview 7 – Olympic Trials and Letting Go**

By her seventh interview, A. had competed at the Olympic trials. She performed better than expected, even winning a match. But she admitted she had not been fully trained or back to her old self. Still, she surprised her coaches and proved to herself she could compete again. What surprised her most was her reaction: for the first time in her career, she felt no extreme emotion. Winning didn’t bring elation, and losing didn’t bring tears. She was simply at peace with the outcome. Reflecting, she realised her rehab had been shaped by both medical restrictions and her own tendency to “play it safe.” Nutrition had also played a role — once she adjusted her diet, her strength gains improved, but it was too late to change the timeline. She admitted she wasn’t back to her previous peak, but she was proud of what she achieved: “Given everything, I’m proud I even got here.” The trials became not just an ending, but a transition point. She accepted that she would not chase another Olympic cycle. With clarity, A. embraced a new chapter. She prepared her résumé, thought about career paths, and envisioned life beyond sport. Wrestling would always be part of her identity, but she no longer felt trapped by it. She found peace in her faith, her health, and the prospect of building a new future.

**E, 26 y, Judoka, hamstring tendon rupture, 1^st^ interview 17 days after surgery. Total of 7 interviews.**

**Interview 1 – Exhaustion, Pain, and Doubt**

Twelve days after surgery, E. sat down for her first interview. At only 26, she had already endured six major surgeries: three on her shoulder, one on her elbow, one on her back, and now one on her leg. She said: ‘It is a difficult road, full of interruptions and painful comebacks.’

She was devastated. Injured during a competition, she admitted she had cried every day since. Her whole life revolved around judo, and each injury felt like a piece of her dream slipping away. The timing made it worse: twice now, she had been in peak form just after the Olympic Games, only to be derailed by another surgery. She wondered aloud whether she had the strength to start again. Sometimes she thought of walking away, but that thought felt like “a part of me is dying.” Her parents, friends, and a mental coach supported her, and she placed great importance on finding trustworthy health professionals. Her surgery was done abroad as she sought for the best specialist.

**Interview 2 – Physically recovering but alone with Doubts**

Seven weeks after surgery, her body was healing well, but her mind was struggling. Watching competitions from the outside was torture; she felt cut off, “alone most of the time.” She described living in “two moods”: one part of her wanting to return, the other part imagining leaving judo behind.

She carried guilt, wondering if she could have prevented this injury or if there was something fundamentally wrong with her body. She tried to avoid depressive thoughts, supported by her mental coach, but the conflict remained sharp. Her strategy became breaking things down into small steps: focusing only on the next physio session, not on Olympic dreams.

**Interview 3 – Progress in Body, Uncertainty in Mind**

By the third interview, rehab was going well: she was pain-free, stronger, and gaining function. But when the doctor postponed her return after reviewing new MRI scans, her mental conflict deepened.

She confessed to feeling like a failure and described herself as sad and lonely. Friends’ opinions added to her confusion: some urged her to keep going, insisting she was strong enough; others, including her parents, gently supported the idea of ending her career. Their fatigue mirrored her own. She admitted she avoided competitions, avoided social media, and avoided reminders of what she was missing. At the same time, she found glimpses of freedom in situations like going to a nightclub, dancing without pain, realising there was life outside sport. For the first time, she allowed herself to say she didn’t need to decide right away.

**Interview 4 – The Decision to Stop**

By the fourth interview, E. reached clarity: she was ready to stop. Too many injuries, too many comebacks, too much fear of what might happen if she pushed again. She spoke with her coach, who agreed. Her parents supported her as well. With no external pressure to continue, she admitted she had “had enough.”

Still, uncertainty lingered. She often spoke of her “body and mind,” acknowledging that her body was tired and her mind exhausted by the cycle of recovery and reinjury. She had not yet fully embraced the finality of her decision, but she knew this was the healthiest choice.

**Interview 5 – Peace with the Decision**

In the fifth interview, E. was more settled. She had spoken with friends and coaches, and the decision to retire brought a measure of peace. She understood that prioritising her health was necessary.

She tested herself by joining a judo training session. She wasn’t afraid in the moment, but she also knew that competing at 100% would be too risky — her body could not handle another injury, and her mind could not withstand the trauma of starting over yet again. She accepted that letting go was not weakness, but wisdom.

**Interview 6 – Living with Doubts**

Despite her decision, the sixth interview revealed how hard the transition could be. Still living at the training centre, surrounded by teammates, she felt trapped between two worlds. She admitted to feeling guilty, second-guessing herself, and fearing future regret. “My body is tired,” she repeated, “and I need to move on.”

She understood her choice was the right one, yet the sadness was profound. She tried to convince herself that life was bigger than judo, but she struggled with the reality of leaving behind the world she had lived in since childhood.

**Interview 7 – Closure and a New Beginning**

Living again in her hometown, E. is feeling better. Distance from the training environment allowed her to see more clearly. She affirmed her decision: it was about health, balance, and the reality of her injuries. Judo was still “beautiful,” but no longer for her.

She was preparing for a new chapter, waiting to settle into her own place and begin a new work. She admitted it was still painful to think about how her career had ended, and she avoided watching the Olympics to protect herself. Yet she recognised she had made the best choice. With the support of her parents and friends, she began to imagine a life beyond the mat. Looking back, she realised she had often been alone in her rehab, lacking support from her physiotherapist or coach and this loneliness had shaped her journey.

**D, 19 y, tennis player, 5^th^ metacarpal fracture, 1^st^ interview 25 days after surgery. Total of 4 interviews.**

**Interview 1 – Shock, Setback, and Uncertainty**

D. has a promising career. As a young professional tennis player, he had already faced the demands of high training loads and was recovering from a long elbow overuse injury. Just as he was regaining confidence, a traumatic injury during training forced him into surgery abroad. The injury came as a devastating blow. He was suddenly unable to compete, stripped of daily routines, and forced into dependency on his parents for even simple tasks.

The emotional toll was immense. D. described feeling frustrated, uncertain, and even fearful of what the future might hold. Conflicting medical opinions made the situation worse, leaving him unsure if he would ever play pain-free again, the prognosis was uncertain. At the same time, he leaned heavily on his support system: family, coaches, and physiotherapists. He tried to cope by establishing routines, engaging in mental training, and holding on to the hope that small steps forward would eventually lead to a comeback.

**Interview 2 – Early Recovery and Fragile Optimism**

By the second interview, recovery is progressing. The surgical wires had been removed, the infection was under control, and he was beginning to reintroduce tennis. At first, he worked with softer balls, gradually progressing toward normal training. Though he still experienced stiffness, swelling, and pain, he was encouraged by the visible weekly progress.

Psychologically, he was shifting. The despair of the first phase gave way to cautious optimism. Seeing improvements motivated him to keep going, though he sometimes wished for clearer medical guidance rather than just being told to “listen to his body.” He used the injury as an opportunity to learn; improving his nutrition, sleep, and recovery habits and to see himself not only as someone fighting through injury but also as an athlete gaining new tools for the future.

**Interview 3 – Return to Competition and Growth**

By the third interview, D. was back on court competing again. The return was challenging at first. He lost conditioning and rhythm making matches difficult, but he regained strength and even improved parts of his game. Tactically, he used his downtime to refine his attacking play. His finger remained a little stiff and required warm-up exercises, but he was functioning at a high level again.

Emotionally, this was a turning point. D. expressed gratitude for the journey, saying he appreciated milestones that once seemed minor such as sweating again, lifting weights, stepping into his first match. What once had been a devastating injury became a source of growth and resilience. He described himself as more patient, disciplined, and mentally strong. Importantly, his perspective shifted: rankings and short-term performance mattered less, while long-term progress and sustainability took priority. He also reflected on how deeply medical communication had affected him, remembering how painful it was to be told he might never play pain-free, and highlighting the importance of hope in the recovery journey.

**Interview 4 – Full Return and a Mature Perspective**

In the final interview, D. reflected with pride on a complete return to competition. He was pain-free, back at his previous level, and confident in his body. The injury no longer felt like a setback but rather a turning point in his career and life.

He explained that he had learned to listen to his body, prioritise health, and avoid pushing himself too hard in a sport where the calendar offers no natural breaks. He now approached training and competition with greater balance and perspective. What could have been a purely negative episode had, in his view, made him a stronger and wiser athlete.

**C, 42 y, volleyball athlete, ACL tear, 1^st^ interview 10 days after surgery. Total of 6 Interviews**

**Interview 1 – Shock and First Reflections**

When C, Olympic medallist with long experience on the national team, suffered her first major injury, it felt like a shock. In a career where she had always been strong and without any major injury, she suddenly found herself on crutches, dependent on others, and unable to train. The injury threatened not only her immediate season but also her carefully imagined retirement plans. She admitted she had not considered quitting, but she felt deeply threatened by the thought that her career might end in a way she had never planned.

Emotionally, the first weeks were heavy. She described feeling mentally tired and even burned out at the time of the injury, sometimes crying and feeling anxious, other times forcing herself to see the positive side. Small victories such as gaining a little range of motion or a step taken more easily became milestones that helped her keep going. She leaned on her family, her trusted physiotherapist, and a mental coach, while keeping alive her dream of returning to the court, maybe even at another Olympics.

**Interview 2 – Motivation Through Progress**

By her second interview, C.’s tone had changed. The visible progress she was making gave her motivation and hope. She described feeling positive about her rehab process, encouraged by the steady improvements she could see and measure. Her trust in her physiotherapist remained a central anchor, providing the reassurance that “everything is being done right.”

Still, recovery wasn’t only physical. Being around her team was difficult; she needed some emotional distance at times. Watching her teammates compete when she could not left her feeling excluded and vulnerable. She acknowledged how much the broader sports environment affected her mental state.

**Interview 3 – Finding Balance and Routine**

In the third interview, C. expressed that she had found a rhythm and balance in her rehabilitation. She had settled into a routine, was still strongly supported by her physiotherapist, and felt surprised and motivated by the constant improvements she was noticing.

Her outlook was more positive and more patient. She had come to understand the process better and accepted that she could not push it any faster. While uncertainty about her long-term future still lingered, she was learning to live with it and trust that waiting was part of the journey.

**Interview 4 – Identity Restored and Personal Growth**

By the fourth interview, C. was running, jumping, and training on the court again. These movements gave her back something she had missed dearly: her identity as an athlete. She said she no longer felt like a patient in rehab, but once again like a volleyball player. The return of her athletic identity was deeply motivating, even though she was still progressing gradually and under controlled conditions.

She admitted she disliked the limits the injury imposed, but also acknowledged the discipline, consistency, and patience required to overcome them. Her Olympic dream remained alive, though she now saw it as more distant, but not impossible. She also reflected on her personal growth. The injury had widened her perspective beyond sport, helping her see herself not just as an athlete but as a human being with broader connections and values.

Reintegration with the team brought another layer of reflection. Early in rehab, she had felt excluded, but now that she was functioning better, she felt part of the group again. Her bond with her physiotherapist also stood out, providing not only technical support but also encouragement, perspective, and space to reconnect with family.

**Interview 5 – Return to Training, Frustrations with Matches Opportunities**

By the fifth interview, C. was fully back in training, doing everything alongside her teammates. She had passed strength testing but still needed to wait the required nine months before being medically cleared to play matches. Being back with the team felt “wonderful,” though she admitted movements didn’t always feel completely natural yet.

However, her excitement was tempered by frustration. The season’s schedule and the club’s priorities meant she would not be reintegrated into matches quickly. She felt less of a priority for the team and sensed that the club was not counting on her for that year. At the same time, fans, sponsors, and media put pressure on her return, making her feel both wanted and insecure.

Despite these tensions, C. described the injury as “one of the best things” that had happened in her life. It gave her patience, empathy, and perspective. It allowed her to spend more time with her family and reflect on her career transition. Rehab had become not only about physical recovery but also about reshaping her identity and imagining life after volleyball.

**Interview 6 – Return, Limitations, and Acceptance**

In her final interview, C. reflected on her comeback. Physically, she had returned without restrictions, proud of her recovery at age 42. She felt ready, not fearful of reinjury, and proud of how far she had come. Yet her return did not play out as she had hoped. Because of team decisions and the presence of a new player in her position, she had few opportunities to play matches. What limited her was no longer her body but the context of the team and organisation.

This lack of opportunities frustrated her and impacted her chance to still participate in the Olympic Games. She longed to show her level and regain her rhythm, but she recognised that these decisions were beyond her control. With maturity, she accepted that she had done everything possible and chose to focus on what she could control: her preparation, her mindset, and her future.

C. finished this stage of her journey with both pride and anticipation. She was proud of the lessons learned: patience, empathy, appreciation of small milestones, and gratitude for being back in team routines. And she looked forward to a new contract with a new club, optimistic that she could still demonstrate her true performance level with the right opportunities.

**J, 19 y, football player, ACL tear, 1^st^ interview 3 days after surgery. Total of 7 interviews.**

**Interview 1 – Injury, Surgery, and Determination**

J., a 19-year-old football player with national team experience and a past in British youth football, entered his first interview shortly after suffering a major setback: an ACL rupture and meniscus injury sustained during a match. The timing was devastating. He had recently won the league, was planning another strong season in his home country, and hoped to return to England to pursue his career abroad. Instead, he found himself facing another surgery after previous meniscus surgery.

Despite the frustration and sadness, J. approached the situation pragmatically. “It’s football, it’s a process,” he said, acknowledging that injuries were part of the game. With privileged access through the national team, he quickly received scans, surgery, and top medical care. His close bond with his previous physiotherapist, along with strong support from his family, girlfriend, and teammates, gave him confidence. He coped by focusing on small daily goals, eating well, and keeping busy with his side job in real estate.

**Interview 2 – Complications and Stress**

The early months of recovery brought unexpected challenges. After surgery, J. developed a thrombosis in his calf, which forced him to rest for nearly two weeks and delayed his progress. Regaining knee flexion and extension became harder, leaving him constantly aware of his injury: “I’m thinking about my knee probably like 24 hours”.

Staying motivated was difficult, particularly as he watched his teammates train and compete while he sat on the sidelines. Attending training kept him connected but also reminded him of what he was missing. He avoided watching matches altogether, describing the pain of seeing the national team play without him.

Tensions arose between the national team and club physiotherapists, who disagreed on his rehabilitation program. This left him stressed and uncertain about which path to follow. Ultimately, J. decided to trust the club physiotherapist he knew well, though he continued navigating the politics of staying aligned with the national setup. He leaned on his real estate work as a mental outlet, but his primary goal remained clear: to recover fully and keep his football ambitions alive.

**Interview 3 – Settling into Rehab and Finding Perspective**

By the third interview, J. had adjusted to the rehabilitation rhythm. About three months post-surgery, he began to see visible improvements: his muscles were strengthening, his knee was responding well, and he even started light jogging. The monotony of gym sessions was still challenging, but progress gave him motivation: “The weeks are passing quickly at the moment.”

He reflected on life outside football, wondering what his future would be if his career did not progress as planned. Working as a property agent gave him distraction and a sense of stability, while his experience with previous injuries helped him maintain perspective. Though he continued to avoid watching competitions to protect himself emotionally, he remained present in the team environment. The earlier disagreements between the physiotherapists (club and national team) had now been resolved.

**Interview 4 – Returning to Football-Specific Training**

The fourth interview marked a turning point. J. had begun football-specific work: running, endurance drills, and skill training with a football coach focused on his position. This gave him a renewed sense of identity: “Now I feel more like an athlete again, I’m getting back.”

He sought reassurance from a former physiotherapist he trusted, who confirmed that his progress was on track and guided him on what to focus on. While he still thought about his knee often, the intensity of worry had lessened. Outside football, real estate work continued to give him perspective and balance.

Still, he was realistic about timing. With the season nearing its end, he knew opportunities for match play would be limited. He anticipated that his true return would likely come during the next preseason, but he was learning to take things day by day.

**Interview 5 – Back with the Team and New Concerns**

By the fifth interview, J. had reintegrated into team training, though under modified conditions that avoided contact. He reported feeling physically well and rarely thought about his knee in daily life or during training. His main concern had shifted to the future: “It’s more about my future that I think about, if I’m capable of getting back to 100%.”

He appreciated the role of clinical testing as a guideline, but emphasised that true readiness could only come through football itself like playing with the ball, in real situations. Training with the team again gave him motivation and a sense of accomplishment, though he acknowledged the challenge of proving himself after such a long absence. He was also in contact with another club that could offer more playing time, reflecting his pragmatic approach to sustaining his career. J. reflected that the injury had changed him: “I think I changed for the better. I grew up from it.”

**Interview 6 – Transitioning Toward Return**

In the sixth interview, J. was training fully but still lacked match experience. With the season finishing and the international break approaching, he recognised that he would not have opportunities to build game rhythm in time to be selected. He described the final phase of recovery as more about game-specific demands such as tactical awareness, match sharpness, and volume of play, aspects that cannot be fully replicated in training.

He also noticed himself relying less on external support and more on his own independence, a sign of maturity and confidence in his ability to navigate his journey. Real estate continued to provide balance, reminding him that life outside football could be meaningful as well.

**Interview 7 – Setbacks, Lessons, and a Broader Outlook**

By the seventh interview, J. had transferred to another club to secure more consistent playing time. However, he suffered a hamstring strain, which he accepted as a minor setback. He saw the injury as part of the learning process, reflecting that previous injuries had taught him resilience and patience.

Although eager to regain game volume and return to his full level, J. also emphasised perspective: “Life is more than football.” The injury had broadened his outlook, teaching him to value balance, growth, and the possibility of alternative paths. Looking toward the next season, he remained ambitious but grounded, taking things day by day and focusing on steady progress both on and off the pitch.
