# Supplementary material for: From ‘My world fell apart’ to ‘The best thing that happened in my life’: elite athletes’ longitudinal journeys after an injury
Source: BMJ Open Sport Exerc Med. 2026 Sep 25;12(3):e003320. doi: 10.1136/bmjsem-2026-003320 (PMC13630066; doi:10.1136/bmjsem-2026-003320)
Supplement: Supplementary file 2 [file bmjsem-12-3-s002.docx]

**Interview guide**

1. Could you give me a short introduction to your career in sports?

2. Could you tell me a bit about the injury and what happened to you?

3. Could you describe how you feel at the moment? Prompt: physically, emotionally, mentally

4. How important to you are your sports career, your team, coach, family, and school? How does this injury and the rehabilitation process affect your everyday life? Prompt: work, interests, relationships.

5. How much do you think about your injury? When you do so- what comes into your mind? What are some of the demands/ needs you think you need to return to sport?

6. Since the injury, do you see a difference in how you see yourself? Prompt: If so, how do you see yourself now as different? How would you say you have changed?

7. How has your experience been so far with the rehabilitation process? Prompt: care, social support? Who is involved in the process at the moment? ( planning decision making,

8. Do you feel like you have had any progress / improvement? If so, tell me about your progress/improvement. What do you consider a milestone in the process so far? Prompt: What do you think of the ways you are being tested/ assessed? Do you think they accurately reflect your progression/ improvement?

9. How are you coping with the process? Which strategies did you develop to deal with the situation at the moment? Prompt: Who is helping you? How did you learn to cope with it?

10. What are you expecting from the process? What do you imagine will be the next steps? Prompt: do you feel you are ready to start this next phase of the process?

Questions 1 and 2 were only asked in the first interview. The other questions were asked in all interviews, with potential new questions added to explore the athlete’s perceptions better.
