# Supplementary material for: From ‘My world fell apart’ to ‘The best thing that happened in my life’: elite athletes’ longitudinal journeys after an injury
Source: BMJ Open Sport Exerc Med. 2026 Sep 25;12(3):e003320. doi: 10.1136/bmjsem-2026-003320 (PMC13630066; doi:10.1136/bmjsem-2026-003320)
Supplement: Supplementary file 3 [file bmjsem-12-3-s003.docx]

| Sport/ age/ sex | Interviews | Dates | Durations | Date of 1^st^ interview | Injury |
| --- | --- | --- | --- | --- | --- |
| Tennis, 19y, male | 4 | 07/02/2024, 27/03/2024, 12/06/2024,  02/09/2024 | 34’32’’/ 27’42’’/25’25’’/25’40’’= 113’19’’ | 25 days after surgery | 5^th^ metacarpal fracture |
| Volleyball, 42, female | 6 | 11/04/2023, 22/05/2023, 03/07/2023,  11/09/2023, 09/11/2023,  04/04/2024 | 33’18’’/35’10’’/29’20’’/35’  09’’/35’28’’27’38’’= 196’ 03’’ | 10 days after ACL reconstruction | ACL |
| Football, 19 y, male | 7 | 13/09/2023, 20/10/2023,  07/12/2023, 07/02/2024,  12/04/2024,  06/06/2024,  23/08/2024 | 23’33’’/29’32’’/24’54’’/23’  52’’/25’35’’/ 28’08’’/30’16’’= 183’50’’ | First interviews 3 days after ACL reconstruction | ACL |
| Judo, 26 y, female | 7 | 04/04/2023,  11/05/2023, 29/06/2023,  11/08/2023,  29/09/2023,  11/12/2023,  15/02/2024 | 40’20’’/23’34’’/43’39’’/34’  52’’/ 45’06’’/36’40’’/37’01’’= 261’12’’ | 17 days after surgery | Hamstring tendon rupture |
| Wrestling, 29, female | 7 | 05/06/2023,  09/07/2023,  03/09/2023,  08/10/2023,  26/11/2023,  11/02/2024,  14/07/2024 | 30’26’’/29’29’’/37’17’’/39’  22’’/36’46’’43’39’’= 216’59’’ | 8 days after ACL reconstruction | ACL |
| Total: | 31 |  | 16 hours 8 minutes 23 seg |  |  |
